# Adolescents have unfavorable opinions of adolescents who use e-cigarettes

**DOI:** 10.1371/journal.pone.0206352

**Published:** 2018-11-07

**Authors:** Karma McKelvey, Lucy Popova, Jessica K. Pepper, Noel T. Brewer, Bonnie Halpern-Felsher

**Affiliations:** 1 Division of Adolescent Medicine, Department of Pediatrics, School of Medicine, Stanford University, Palo Alto, California, United States of America; 2 School of Public Health, Georgia State University, Atlanta, Georgia, United States of America; 3 Department of Health Behavior, Gillings School of Global Public Health, University of North Carolina, Chapel Hill, North Carolina, United States of America; 4 RTI International, Research Triangle Park, North Carolina, United States of America; University of California San Diego School of Medicine, UNITED STATES

## Abstract

**Introduction:**

While evidence suggests positive opinions of smokers are associated with tobacco use, research exploring adolescents’ opinions of e-cigarette users is nascent. We hypothesized that adolescents harbor positive opinions of e-cigarette users, and that these opinions will be more positive among adolescents willing to try or who have used e-cigarettes.

**Methods:**

Participants were 578 U.S. adolescents (ages 14 to 20) recruited from ten California schools. An online survey assessed their attitudes toward and opinions of adolescents who use e-cigarettes in 2015–2016. Analyses examined whether these variables were associated with willingness to try and use (ever vs. never) of e-cigarettes.

**Results:**

The majority (61%) of participants had negative overall opinions toward adolescent e-cigarette users. Few participants ascribed positive traits (i.e., sexy, cool, clean, smart, and healthy) to e-cigarette users. Participants who were willing to try or had used e-cigarettes endorsed positive traits more than those unwilling to try and never-users (all *p* < .01). Participants sometimes endorsed negative traits (i.e., unattractive, trashy, immature, disgusting, and inconsiderate) to describe e-cigarette users. Unwilling and never-users viewed negative traits as more descriptive of e-cigarette users than willing or ever-users (all *p* < .01).

**Conclusions:**

Adolescents generally had somewhat negative opinions of other adolescents who use e-cigarettes. Building on adolescents’ negativity toward adolescent e-cigarette users may be a productive direction for prevention efforts, and clinicians can play an important role by keeping apprised of the products their adolescent patients are using and providing information on health effects to support negative opinions or dissuade formation of more positive ones.

## Introduction

The 2016 U.S. Surgeon General’s report [1] raised serious concerns about adolescents’ use of e-cigarettes, also known as vapes, vape pens, vaporizers, mods, and tank systems [1]. In national surveys, use of e-cigarettes at least once in the past 30 days among middle- and high-school students was higher (6.2% and 12.5%, respectively) than for use of cigarettes (2.6% and 10.5%) or any other tobacco product [2]. The higher prevalence of e-cigarette use among adolescents may be due in part to more favorable attitudes towards e-cigarettes compared to cigarettes [3–5]. Studies of e-cigarette-related attitudes and perceptions show that adolescents hold more favorable attitudes toward and attribute fewer health and social risks to e-cigarettes compared to other tobacco products [5–8], and that those who have used e-cigarettes hold more positive attitudes towards e-cigarettes [6,9].

People are more likely to be willing to smoke and to actually smoke if they hold more positive mental images or prototypes of a typical smoker [10–14]. Among adolescents who have never smoked, positive prototypes (e.g., cool, sociable, and intelligent) are associated with greater susceptibility to smoke, and those who report having a self-image similar to that of their reported smoker-image (“prototype”) are almost twice as likely to report smoking uptake one year later [10,15]. Positive appraisals of smokers are influenced by best friend’s smoking, outweigh weaker parental antismoking expectations, and lead to greater smoking intentions [16]. Some evidence also suggests that cigarette smoker prototypes are related to e-cigarette use, and that among nonsmoking adolescents who held negative beliefs about a typical smoker, those with scores above the median (compared to those at or below the median score) are less likely to report willingness to try e-cigarettes (24% vs. 12%, *p* = .02) [17].

While ample evidence shows that cigarette smoker prototypes are associated with cigarette use intentions and behaviors [10–16], little research has explored e-cigarette user prototypes among adolescents. Such knowledge is important because messages aimed at countering positive imagery and glamorous portrayals of e-cigarette users could be a promising approach to preventing adolescent e-cigarette use. Our study sought to characterize adolescents’ prototypes of adolescent e-cigarette users and to determine whether these prototypes are related to willingness to use e-cigarettes and actual e-cigarette use [17]. We hypothesized that adolescents who are willing to try e-cigarettes or have used them in the past will hold more positive opinions of e-cigarette users than those who are unwilling to use or have never used them.

## Methods

### Participants

We recruited a convenience sample from ten large high schools in California, US that had diverse populations with respect to race/ethnicity and socioeconomic status. Study personnel visited each of the 9^th^ and 12^th^ grade classes in the ten schools and invited all students in those classes to participate in a study of perceptions, social norms, marketing, and use patterns associated with tobacco products. Students were given study information and consent forms to share with their parents. Interested participants signed assent forms and returned signed parental consent forms. Study personnel returned to the schools a few days later, collected forms, and answered any questions [5].

Overall, 4,246 students were reached by personnel; 1,299 were recruited and consented; and 786 completed the baseline survey in 2013–14. Data used in this study were collected from July 2015 to April 2016 (*n* = 578) and constitute Wave 3 of data collection. Details of the ongoing cohort study are published elsewhere.

### Procedures

Participants completed an online survey administered by Qualtrics (Provo, UT). Instructions encouraged participants to complete the survey all at once, although they could return to it later if needed. Participants received $15 for completing the survey. Stanford University’s institutional review board approved all procedures.

### Measures

#### Demographics

The survey assessed participants’ demographic characteristics including age, sex, Hispanic ethnicity, and race.

#### Use of e-cigarettes

The survey assessed e-cigarette use by asking, “During your entire life how many times have you ever used an e-cigarette/vape, even 1 or 2 puffs?” The response scale options were: never, 1–2 times, 3–10 times, 11–19 times, 20–30 times, 31–99 times, and 100 or more times. We classified those who answered “never” as “never users” and all others as “ever users” [18].

#### Opinions of e-cigarette users

The Opinions of E-cigarette users and the Prototypes items were adapted from the Prototypes of Tobacco Users Scale and items about general, negative, and positive opinions of e-cigarette users (scale and items developed by Drs. Pepper and Brewer at the University of North Carolina, originally used with adults but adapted for adolescents and young adults for this study). We piloted the survey with a small group of students before dissemination.

Three survey items assessed overall opinions about e-cigarette users. Text before the items read: “We’d like to know more about what you think about e-cigarette/vape users IN GENERAL. The following questions are not about you or specific people you know.” The **overall opinion** item read: “Picture a typical e-cigarette/vape user your age. Is your opinion of this person …” The response scale was “very negative” (coded as 1), “somewhat negative” (2), “neutral” (3), “somewhat positive” (4), “very positive” (5).

The **positive opinion** item read: “Think about only your positive opinions of typical e-cigarette/vape users your age, ignoring any negative opinions. How positive are your positive opinions of e-cigarette users? The response scale was “not at all positive” (coded as 1), “slightly positive” (2), “quite positive” (3), and “extremely positive” (4).

The **negative opinion** item read: “Think about only your negative opinions of typical e-cigarette/vape users your age, ignoring any positive opinions. How negative are your negative opinions of e-cigarette/vape users?” Participants chose between “not at all negative” (coded as 1), “slightly negative” (2), “quite negative” (3), and “extremely negative” (4).

#### Prototypes

The prototype items asked participants to determine, “How much do the following characteristics describe a typical E-cigarette/vape user your age,” followed by the terms: sexy, cool, clean, smart, healthy, attractive (positive traits) and trashy, immature, disgusting, and inconsiderate (negative traits). Participants assessed the extent to which each term described their image of a typical e-cigarette user, using the scale: “not at all” (coded as 1), “a little bit” (2), “somewhat” (3), “quite a bit” (4), and “very much” (5).

#### Willingness to try e-cigarettes

The survey assessed willingness to try e-cigarettes by asking, “If one of your friends were to offer you an e-cigarette/vape, would you TRY it?” Response options were “definitely not,” “probably not,” “probably yes,” and “definitely yes.” We dichotomized responses into “not willing to try” (definitely not) and “willing to try” (all other responses) [19].

### Data analysis

Due to the nested nature of the data, differences in outcome measures between groups (schools) were tested for using multi-level mixed modeling. No consistent nor meaningful between-group differences were found. We calculated means for attitudes and images of adolescent e-cigarette-users. Independent samples *t*-tests compared mean differences for each attitude and image by past e-cigarette use (ever vs. never) and willingness to try e-cigarettes (willing vs. unwilling). Analyses were conducted using SPSS 23, using *p* < .05 as the criterion for statistical significance.

## Results

### Demographics

Two thirds of participants identified as female (*n* = 372, 64.4%), and the mean age was 16.7 (SD = 1.7; range = 14 to 20). Participants were ethnically diverse (*n* = 217, 38.5% Hispanic) and racially diverse, with 214 who identified as “white” (39.4%), 129 (23.8%) as Asian, 122 (22.5%) as “more than one race,” and 78 (14.4%) other.

### E-Cigarette use

Among the participants, n = 422 (73%) never used, 66 (11.4%) used e-cigarettes 1–2 times, 39 (6.7%) used e-cigarettes 3–10 times, 19 (3.3%) used 11–19 times, 9 (1.6%) used 20–30 times, 9 (1.6%) used 31–99 times, and 12 (2.1%) used 100 or more times; 2 people (0.3%) didn't respond to the question.

### Opinions of adolescent e-cigarette users

**Fig 1:**
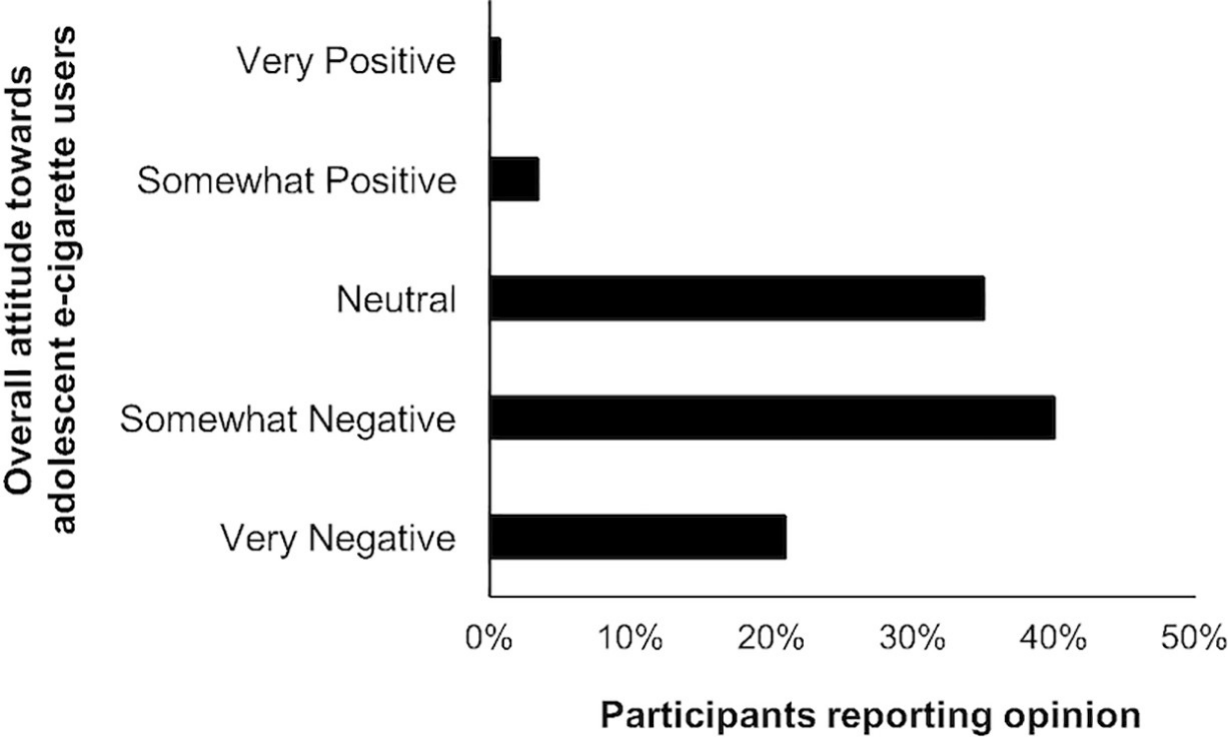
Overall opinions toward adolescent E-cigarette users (*n* = 578).

Participants’ overall opinion toward the typical adolescent e-cigarette user was somewhat negative, with an average score of 2.23 out of 5 (between “somewhat negative” and “neutral”) (Table 1). The majority (61%) of participants indicated somewhat or very negative overall opinions of e-cigarette users (Fig 1). Only 4% of participants had either somewhat or very positive overall opinions of adolescent e-cigarette users. Participants not willing to try e-cigarettes and never users of e-cigarettes reported more negative overall opinions than adolescents who were willing to try or who had used e-cigarettes (*p* < .001; Table 1).

**Table 1:** Mean (SD) overall, positive, and negative opinions toward adolescent E-cigarette users, for full sample and separately for those not willing and willing to try E-cigarettes, and for never and ever users of E-cigarettes.

Opinions	Full Sample	Willingness to Use E-cigarettes			Past Use of E-cigarettes		
	(*n* = 578)	*Not Willing* (*n* = 321)	*Willing* (*n* = 256)	p-value*	*Never Used* (*n* = 421)	*Ever Used* (*n* = 154)	p-value*
**Overall**^*a*^	2.22 (0.9)	1.96 (0.8)	2.55 (0.8)	**< .001**	2.09 (0.8)	2.58 (0.8)	**< .001**
**Positive attitude**^*b*^	1.66 (0.7)	1.49 (0.6)	1.88 (0.7)	**< .001**	1.60 (0.7)	1.84 (0.7)	**< .001**
**Negative attitude**^*c*^	2.64 (0.9)	2.88 (0.9)	2.34 (0.9)	**< .001**	2.79 (1.0)	2.27 (0.8)	**< .001**

SD = standard deviation.

*Independent samples *t-*test

^a^Response scale ranged from “very negative” (coded as 1) to “very positive” (5).

^b^Response scale ranged from “not at all positive” (coded as 1) to “extremely positive” (4).

^c^Response scale ranged from “not at all negative” (coded as 1) to “extremely negative” (4).

**Fig 2:**
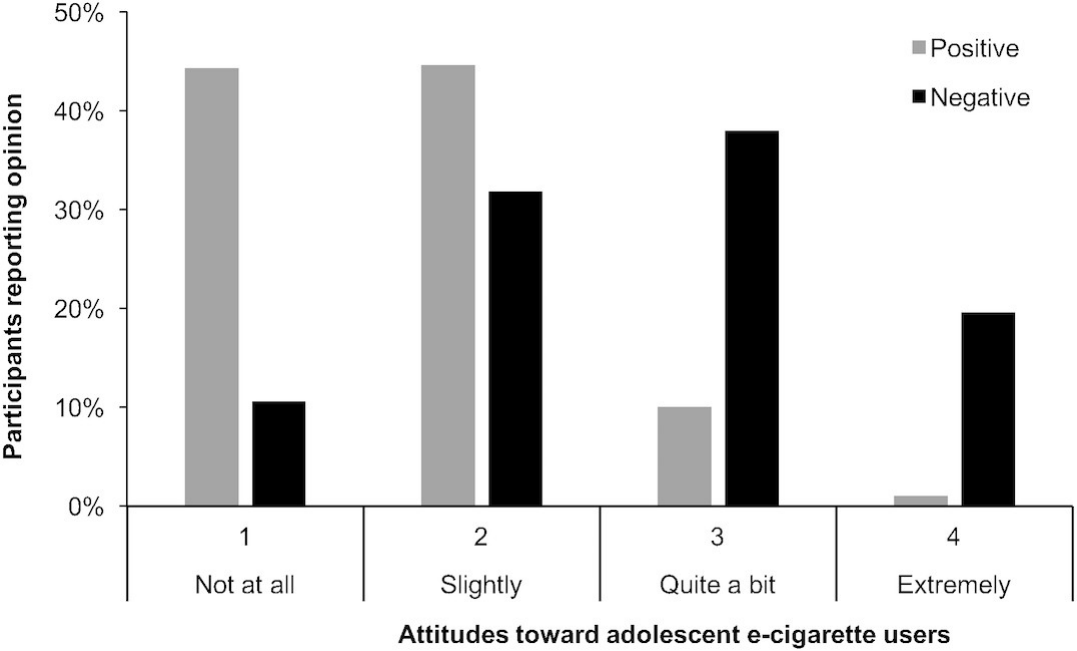
Positive and negative opinions about adolescent E-cigarette users (*n* = 578).

When asked to consider only their positive or negative opinions, adolescents reported weaker positive opinions (mean 1.67 on a unipolar response scale coded as 1 to 4) than negative opinions (mean 2.65) (Table 1). Almost 90% of the sample reported opinions about adolescent e-cigarette users that were not at all positive or only slightly so, and 59% reported opinions about e-cigarette users that were quite a bit or extremely negative (Fig 2). Those not willing to try e-cigarettes and never-users of e-cigarettes reported weaker positive opinions and stronger negative opinions regarding adolescent e-cigarette users than those who were willing to try or had ever used e-cigarettes (Table 1).

### Prototypes of adolescent e-cigarette users

Participants infrequently described adolescent e-cigarette users using positive traits (sexy, cool, clean, smart, and healthy). Positive traits received ratings of “not at all” to “somewhat” on average (means ranged from 1.30 to 1.68; Table 2). Endorsement of positive traits was stronger among adolescents who were willing to try or had used e-cigarettes in the past than those unwilling to try and who never used e-cigarettes (all *p* < .01). In contrast, participants sometimes described adolescent e-cigarette users using negative traits (unattractive, trashy, immature, disgusting, and inconsiderate). Negative traits received ratings of “a little bit” to “somewhat” on average (means ranged from 2.74 to 3.02; Table 2). Endorsement of negative traits was stronger among adolescents unwilling to try and never users of e-cigarettes than those who were willing to try or had used e-cigarettes (all *p* < .01).

**Table 2:** Mean (SD) traits ascribed to adolescent E-cigarette users, by full sample, by not willing and willing to try E-cigarettes, and by never and ever users of E-cigarettes.

		Willingness to Use E-cigarettes			Past Use of E-cigarettes		
Traits	Full Sample (*n* = 578)	Not Willing (*n* = 321)	Willing (*n* = 256)	*p-*value*	Never Used (*n* = 421)	Ever Used (*n* = 154)	*p-*value*
*Positive*							
Sexy	1.30 (0.77)	1.16 (0.52)	1.50 (0.98)	< .01	1.23 (0.64)	1.52 (1.00)	< .01
Cool	1.49 (0.93)	1.31 (0.81)	1.73 (1.03)	< .01	1.41 (0.87)	1.70 (1.04)	< .01
Clean	1.68 (1.00)	1.50 (0.85)	1.92 (1.13)	< .01	1.55 (0.88)	2.04 (1.18)	< .01
Smart	1.58 (0.90)	1.45 (0.82)	1.74 (0.99)	< .01	1.49 (0.82)	1.81 (1.07)	< .01
Healthy	1.58 (0.96)	1.46 (0.86)	1.76 (1.05)	< .01	1.50 (0.89)	1.82 (1.07)	< .01
*Negative*							
Unattractive	3.02 (1.55)	3.44 (1.50)	2.44 (1.41)	< .01	3.29 (1.52)	2.32 (1.37)	< .01
Trashy	2.81 (1.48)	3.12 (1.49)	2.38 (1.36)	< .01	3.00 (1.47)	2.32 (1.37)	< .01
Immature	3.02 (1.49)	3.33 (1.49)	2.59 (1.41)	< .01	3.20 (1.49)	2.55 (1.34)	< .01
Disgusting	2.80 (1.49)	3.23 (1.48)	2.23 (1.30)	< .01	3.08 (1.49)	2.10 (1.23)	< .01
Inconsiderate	2.74 (1.39)	2.96 (1.44)	2.44 (1.27)	< .01	2.88 (1.41)	2.39 (1.26)	< .01

SD = standard deviation

*Independent samples *t-*test

*Note*. Response scale ranged from “not at all” (coded as 1) to “very much” (5).

## Discussion

Numerous studies have described prototypes of cigarette smokers held by adolescents, with more positive images associated with greater likelihood of cigarette use.[10–16] In our study, we extended these findings to examine images of e-cigarette users held by adolescents. Participants reported “somewhat negative” overall opinions of adolescent e-cigarette users, on average. When asked separately about negative and positive opinions, adolescents rated their opinions about e-cigarette users, on average, as “quite a bit” negative and “slightly” positive. Also, the traits unattractive, trashy, immature, disgusting, and inconsiderate received ratings of “somewhat” attributable to adolescent e-cigarette users, on average, while the traits sexy and cool received ratings of “not at all” attributable; and clean, smart, and healthy were “a little bit” attributable. These findings do not support our hypothesis that participants would harbor positive images of adolescent e-cigarette users, which we surmised based on the increase in e-cigarette use over the past few years, perceptions that e-cigarettes are less harmful than other tobacco products, and concerns that e-cigarettes are becoming more socially acceptable [5,7,8,20].

Our findings support our hypothesis that participants who were willing to try e-cigarettes or who had already done so would have more positive prototypes and would hold more positive opinions and images of youth who use e-cigarettes. Research on smoker prototypes and willingness has shown that the more positively the prototypical smoker is seen, the more willing children and adolescents are to smoke [13,21]. Our findings supported our hypothesis that prototypes are associated with past e-cigarette use. Other research has shown that opinions and images may change to support the behavior, once begun [14,22]. We could not determine such relationships with our cross-sectional data; future study should examine these prospective relationships.

Evidence shows pervasive marketing of e-cigarettes targets adolescents by including attractive models, appealing flavors, and claims of lower harm [23–27], which can lead to formation of positive images of people who use e-cigarettes [28–31]. However, our findings suggest these messages have not translated to positive images of e-cigarette users among this group of adolescents. It is important to ensure that adolescents do not formulate “cool” or otherwise desirable images of e-cigarette users, as these images could become established, as occurred with the positive cigarette-smoking prototypes, which took decades of concentrated efforts to dispel [32]. Since most children begin and adolescents continue to build their prototypes based on what they see in mass media, prevention efforts could include media campaigns that counteract creation of positive imagery by increasing adolescents’ intentions to protect themselves from harm and could include highlighting negative health effects of e-cigarettes and deceptive e-cigarette marketing strategies employed by the tobacco industry [33–36].

Study limitations include use of a convenience sample of high school students in California who may not generalize to other populations; for example, in places where prevalence of adolescent e-cigarette use is much higher, adolescents may hold more positive opinions and have more positive images. The cross-sectional nature of the study did not allow us to determine whether the prototypes drive behavior or vice versa. Longitudinal studies should prospectively examine whether prototypes at baseline predict behavior at follow-up, and intervention studies could attempt to manipulate prototypes to change tobacco use behavior. The data were collected in 2015–2016, before the rise of popularity in JUUL electronic cigarettes [37]. Nonetheless, these findings are useful for the purpose of historical comparisons and for potentially understanding the current context of e-cigarette use. For example, future studies might examine how user prototypes have changed with the rise of JUUL.

In this study we did not differentiate between perceptions of adolescents who use e-cigarettes with or without nicotine. While many adolescents report that they vape non-nicotine substances (i.e., “just flavors”) [38] adolescents who self-report using e-cigarettes without nicotine have less knowledge about e-cigarettes, indicating they might not be correctly understanding and reporting their nicotine [39]. Furthermore, a recent chemical analysis of e-cigarettes confiscated in Arizona schools showed that nearly 80% of them contained nicotine, and 57% contained very high levels of nicotine (over 44 mg/mL) [Mansur, E. 2018. E-Liquid Lab Testing and School Resource Officer Outreach in Arizona. Presented at the Public Health Law Center Webinar “What’s the hype? JUUL electronic cigarette’s popularity with youth & young adults.” Available at http://www.publichealthlawcenter.org/webinar/what%E2%80%99s-hype-juul-electronic-cigarette%E2%80%99s-popularity-youth-young-adults)]. Thus, adolescents are frequently incorrect in their assessment of the nicotine content of the substance they are vaping. Future studies should evaluate whether adolescents’ opinions of vapers differ depending on the substance they are vaping.

Our study found that adolescents typically have negative opinions about and images of adolescents their age who use e-cigarettes. However, adolescents who were willing to use e-cigarettes or who reported already having used e-cigarettes held more positive opinions about and images of vapers their age. These findings suggest promising avenues for reducing e-cigarette use could include monitoring prototypes, restricting advertising that leads to more positive prototypes, and developing public health messaging campaigns that counter positive images and reinforce negative opinions without directly stigmatizing the users (perhaps by revealing industry involvement) [1]. A challenge for communication campaigns is as the e-cigarette marketplace grows and changes so will opinions and images of e-cigarette (and other tobacco products) users. Ongoing research can help public health efforts be attuned to changes in opinions, and longitudinal studies should prospectively examine whether prototypes predict later tobacco use behavior. Regulatory efforts that deal with perceptions of e-cigarette users can also be informed by past tobacco efforts and research that dealt with perceptions of cigarette smokers in the media [40–42]. Clinicians can play an important role by keeping apprised of what type of e-cigarettes their adolescent patients are using, how patients who are non-users view those who use e-cigarettes, and by providing accurate information on the health-effects of e-cigarettes to support existing negative images and to dissuade further use and positive image development. In sum, although it is encouraging that many adolescents already hold somewhat negative beliefs about adolescents who use e-cigarettes, more can be done with this information to prevent initiation or encourage cessation of e-cigarette use.
